# Contribution of Water, Sanitation, and Hygiene Facilities to Maternal Hygiene Practices During Complementary Feeding in Ethiopia: A Systematic Review and Meta‐Analysis

**DOI:** 10.1002/hsr2.73084

**Published:** 2026-08-26

**Authors:** Negasa Eshete Soboksa, Dinkinesh Begna Gudeta, Renay Helouise Van Wyk, Zoe Nomakhushe Nxusani, Vhushavhelo Nedzingahe, Xikombiso Gertrude Mbhenyane

**Affiliations:** ^1^ Division of Human Nutrition, Faculty of Medicine and Health Sciences Stellenbosch University Cape Town South Africa; ^2^ Department of Environmental Health, College of Health Sciences and Medicine Dilla University Dilla Ethiopia; ^3^ Department of Nursing, College of Health Sciences Arsi University Asalla Ethiopia; ^4^ Department of Environmental Health, Faculty of Health Sciences University of Johannesburg Johannesburg South Africa

**Keywords:** complementary feeding, Ethiopia, hygiene facility, maternal hygiene, sanitation accessibility, Water supply

## Abstract

**Background and Aims:**

Complementary feeding is essential for child growth and development, and good maternal hygiene practices are crucial for preventing infections. In Ethiopia, inadequate water, sanitation and hygiene (WASH) facilities can compromise food safety, increasing the risk of diarrhoea and undernutrition. This systematic review and meta‐analysis aim to evaluate the contribution of WASH facilities to maternal hygiene practices during complementary feeding in Ethiopia.

**Methods:**

Relevant studies were identified through searches of PubMed, Scopus, Web of Science, JBI, the Cochrane Library, Google Scholar, and the reference lists of eligible articles. The search strategy was developed using the Population–Exposure–Outcome framework and included terms related to WASH, maternal hygiene practices, and complementary feeding in Ethiopia. Studies were screened against predefined inclusion and exclusion criteria. Data was extracted, charted, and synthesized to summarize evidence on the contribution of WASH facilities to maternal hygiene practices during complementary feeding.

**Results:**

Twelve studies were included in the review. The prevalence of good maternal hygiene practices ranged from 22.2% to 62.0%. Most participants used piped or protected water sources for drinking water. Access to latrines varied from 2.4% to 96.4%, while the availability of handwashing facilities ranged from 1.4% to 64.0%. Meta‐analysis showed that mothers with access to piped or protected water sources were more likely to practise good hygiene during complementary feeding (POR = 3.41; 95% CI: 1.50‐7.78). Positive associations were also observed for access to latrines (POR = 1.70; 95% CI: 0.79–3.66) and handwashing facilities (POR = 2.20; 95% CI: 1.03–4.69).

**Conclusion:**

Although the availability of WASH facilities varied across studies, the findings consistently indicated that access to safe water, sanitation, and handwashing facilities improves maternal hygiene practices during complementary feeding. Expanding WASH infrastructure and integrating hygiene education are essential to promote safer feeding practices and improve child health outcomes in Ethiopia.

## 1 Introduction

Maternal hygiene practices during complementary feeding are essential for protecting the health and well‐being of infants and young children, particularly in resource‐constrained settings such as sub‐Saharan Africa [1, 2, 3, 4]. Complementary feeding, defined as the introduction of solid, semi‐solid, or soft foods alongside continued breastfeeding, represents a critical period in child development during which nutritional requirements increase substantially to support optimal growth and development [5]. The World Health Organization (WHO) recommends the introduction of complementary foods from 6 months of age, with continued breastfeeding up to 23 months and beyond, as infants at this stage are generally physiologically and neurologically prepared to consume foods in addition to breast milk [6]. Complementary foods are intended to bridge the gap between a child's nutritional requirements and the nutrients provided by breast milk alone. These foods may comprise household foods adapted for young children or specially formulated transitional foods designed to meet their specific nutritional needs [7].

Practising appropriate handwashing with soap and maintaining good hygiene during the preparation and feeding of complementary foods can substantially reduce the transmission of pathogenic microorganisms, thereby lowering the incidence of diarrheal disease, acute respiratory infections, and skin infections among young children [8]. However, in sub‐Saharan Africa, including Ethiopia, infants and young children aged 6 to 23 months, particularly those experiencing moderate acute malnutrition, remain highly vulnerable to inadequate dietary intake and poor hygiene conditions, which contribute to increased morbidity and mortality [9]. Inadequate maternal hygiene practices during complementary feeding heighten the risk of food and water contamination, increasing children's exposure to infectious diseases and exacerbating the burden of diarrheal diseases [1, 2]. Frequent episodes of diarrhoea can impair nutrient absorption, reduce appetite, cause dehydration, and ultimately contribute to undernutrition, poor growth, and developmental delays [10].

In many low‐ and middle‐income countries, including Ethiopia, contamination of complementary foods remains a significant public health concern. This is largely attributable to unsafe water supplies, poor personal and domestic hygiene, inadequate cleaning of feeding utensils, improper food storage practices, and unsanitary household environments [11]. The problem is further compounded by limited awareness of safe food‐handling practices and inadequate access to essential facilities such as improved sanitation and suitable food preparation areas [12]. Nevertheless, evidence indicates that improvements in WASH infrastructure, particularly reliable access to safe water and adequate sanitation facilities, can promote better hygiene behaviors and reduce the risk of food contamination. Strengthening hygiene practises during complementary feeding, therefore, represents a key intervention for preventing infection and supporting the health, growth, and development of infants and young children [13].

Socioeconomic, educational, and environmental factors play a crucial role in shaping maternal hygiene practices during complementary feeding. Studies from India [14], Kenya [15] and Ethiopia [8, 16, 17] have shown that higher maternal education, better socioeconomic conditions, urban residence, and access to hygiene facilities are associated with improved food hygiene and feeding practices. Furthermore, the availability of safe water, appropriate food storage, modern cooking facilities, and positive knowledge and attitudes towards hygienic feeding have been linked to better hygiene behaviors among caregivers [8, 16, 17]. These findings highlight the need for integrated interventions that combine hygiene education with improved water, sanitation, and household infrastructure to support safe complementary feeding practices.

Despite the well‐established importance of maternal hygiene during complementary feeding, the contribution of WASH facilities to maternal hygiene practices in Ethiopia remains insufficiently understood. The available evidence is fragmented, and to date, no systematic review and meta‐analysis has comprehensively examined this relationship. Addressing this evidence gap is critical for informing targeted interventions and policies aimed at improving infant and young child nutrition and health outcomes. Therefore, this systematic review and meta‐analysis assessed the contribution of WASH facilities to maternal hygiene practices during complementary feeding in Ethiopia, identified existing knowledge gaps, and synthesized the available evidence. The review was guided by the following research question: “*What is the contribution of WASH facilities to maternal hygiene practices during complementary feeding in Ethiopia?”* By addressing this question, the study seeks to provide evidence‐based insights to support program development, policy formulation, and strategies aimed at improving maternal hygiene practices and child health outcomes in Ethiopia.

## 2 Methods

This systematic review and meta‐analysis were undertaken to synthesize the available evidence on maternal hygiene practices during complementary feeding and to examine the contribution of WASH facilities in Ethiopia. The review sought to address the research question comprehensively by collating and critically appraising findings from relevant studies. Methodological transparency and rigor were ensured through adherence to the Preferred Reporting Items for Systematic Reviews and Meta‐Analyses (PRISMA) guidelines (SM 3).

### 2.1 Searching Strategy and Sources

A three‑stage search strategy was developed using the Population–Exposure–Outcome (PEO) framework. First, titles and abstracts were screened to identify relevant keywords and search terms. Second, a comprehensive database search was undertaken based on the following PEO criteria: Population—mothers of children aged 6–24 months in Ethiopia; Exposure – access to and use of WASH facilities; Outcome–maternal hygiene practices during complementary feeding. The full PubMed search strategy is provided in Supporting Material 1 (SM1). Search was conducted across PubMed/MEDLINE, Scopus, Web of Science, the JBI Evidence Synthesis Database, the Cochrane Library, and Google Scholar. Database‑specific search strategies were developed with input from research experts and incorporated relevant subject headings and index terms. Third, the reference lists of all included studies were manually reviewed to identify additional eligible articles not captured through the database searches. This review included peer‑reviewed, full‑text studies conducted in Ethiopia and published between January 2000 and April 2025.

### 2.2 Eligibility Criteria

This review included peer‑reviewed studies that examined the contribution of WASH facilities to the improvement of maternal hygiene practices during the complementary feeding period in Ethiopia, a country characterized by diverse cultural, socio‑economic, and geographic contexts. Eligible study designs comprised quantitative studies, mixed‑methods studies, observational studies, and intervention or experimental studies. Studies were excluded if they lacked sufficient data for analysis or were reviews, case reports, conference abstracts, editorials, or letters to the editor. Further exclusions applied to studies that did not focus on maternal hygiene practices during the complementary feeding period (6–24 months) in relation to WASH facilities or that were conducted outside Ethiopia.

### 2.3 Study Selection

All retrieved records were imported to Mendeley Desktop for duplicate removal. Title and abstract screening were conducted by one reviewer to exclude clearly irrelevant studies. The full text of all potentially eligible documents was retrieved and independently assessed for inclusion by two reviewers. Discrepancies were resolved through discussion with a third reviewer. Reasons for exclusion were documented, and the study selection process was illustrated using a PRISMA flow diagram.

The review focused on mothers in the postpartum period who were actively practising complementary feeding, which typically spans from 6 to 24 months of age, and their WASH facilities. Maternal hygiene practices, the review's primary focus, encompassed handwashing before food‐related activities, safe food storage, clean utensils, and general household sanitation. The secondary outcome examined the contribution of WASH facilities to maternal hygiene practices during complementary feeding. This review explored how access to clean water, sanitation infrastructure, and hygiene resources influenced mothers' ability to support hygiene.

### 2.4 Data Extraction

Following the application of the inclusion and exclusion criteria, data were extracted from the included studies by two independent reviewers (N.E.S. and D.B.G.) using a data extraction form developed in Microsoft Excel. Extracted data were cross‐checked for accuracy to ensure consistency and completeness. The form includes study characteristics (first author, year of publication, region in country, study design, and sample size); WASH facilities characteristics (source of drinking water and household water treatment practices, presence of and type of latrine facility, presence of handwashing and handwashing practice, and kitchen information for food preparation and storage); outcome measures (magnitude of maternal hygiene practices during complementary feeding); and statistical analysis (measures of association (odds ratios and relative risks) between hygiene practices and WASH facilities, along with their 95% confidence intervals and p‐values) and conclusion of the study findings, which were among the data items extracted from each study for this review.

### 2.5 Critical Appraisal

The methodological quality of all included studies was independently assessed by two reviewers (N.E.S. and D.B.G.) using a modified Joanna Briggs Institute (JBI) critical appraisal checklist [18]. The appraisal tool comprised eight criteria: (1) clearly defined inclusion criteria; (2) detailed description of the study participants and setting; (3) valid and reliable measurement of exposure; (4) use of objective, standard criteria for measuring the condition; (5) identification of potential confounding factors; (6) reporting of strategies to address confounding factors; (7) valid and reliable measurement of outcomes; and (8) use of appropriate statistical analyses. Each criterion was scored as either fulfilled (1 point) or not fulfilled (0 points), yielding a total quality score ranging from 0 to 8. Studies were categorized as having low (0–4), moderate (5–6), or high (7–8) methodological quality. Any discrepancies between reviewers were resolved through discussion and consensus, with consultation from a third reviewer (X.G.M.) where necessary.

### 2.6 Data Analysis

The included studies were synthesized according to maternal hygiene practices during complementary feeding, child health outcomes, study settings, methodologies, and key findings, thereby highlighting the evidence linking maternal hygiene behaviors to child health outcomes in Ethiopia. Findings from diverse study types were integrated using narrative and numerical summaries, thematic analysis of qualitative data, and summary statistics from participatory and observational studies. For the meta‑analysis, data were extracted in Microsoft Excel and analysed in STATA (version 19.5). Heterogeneity was assessed using the Cochrane *Q*‑test and *I* ^2^ statistic, with values ranging from 0% to 100% indicating minimal (0%–25%), low (25%–50%), moderate (50%–75%), or substantial (75%–100%) heterogeneity. The pooled effect of WASH facilities on maternal hygiene practices during complementary feeding was estimated using a random‐effects model with restricted maximum likelihood (REML) estimation to account for substantial between‐study heterogeneity. Pooled effect estimates were presented as odds ratios (ORs) with corresponding 95% confidence intervals (CIs) and visualized using forest plots. Subgroup analyses based on the geographical location of the studies were conducted to explore potential sources of heterogeneity in effect estimates. Furthermore, sensitivity analyses were performed to evaluate the robustness of the findings and the influence of individual studies on the overall estimates.

## 3 Results

### 3.1 Search Results

**Figure 1:**
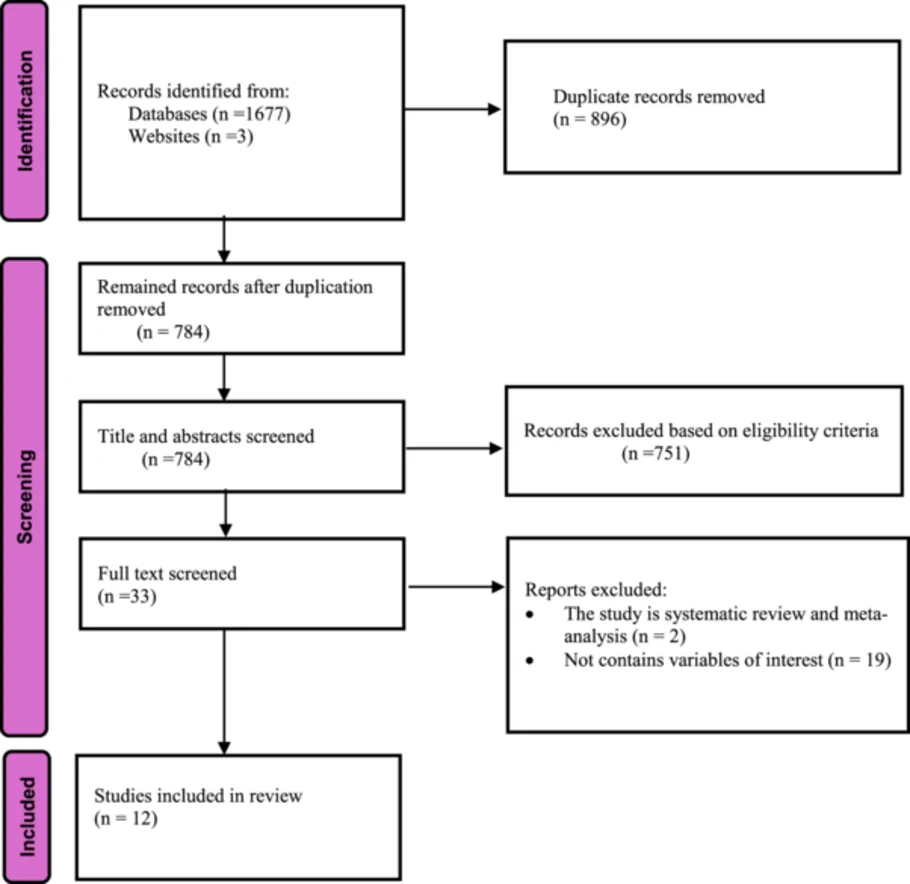
PRISMA flow chart.

After the removal of duplicates, 784 records were screened based on their titles and abstracts, leading to the exclusion of 751 studies. Most records were excluded at this stage because they were not relevant to either WASH facilities and/or maternal hygiene practices during complementary feeding in the Ethiopian context. Subsequently, 33 records were retrieved for full‐text assessment. Of these, two studies were excluded because they were systematic reviews and meta‐analyses, while a further 19 were excluded as they did not address the variables of interest. Ultimately, 12 peer‐reviewed articles met the eligibility criteria and were included in this review (Figure 1).

### 3.2 Description of Included Studies

An overview of the key characteristics of the 12 studies included in this review is presented in Table 1. The studies were conducted across several regions of Ethiopia, including four in the Amhara Region [16, 19, 20, 21], three in Oromia [17, 22, 23], and one each in Central Ethiopia [24], Harari [25], Tigray [8], Southern Ethiopia [26], and Addis Ababa [27]. Of the 12 included studies, one was an institution‐based cross‐sectional study [19], while the remaining 11 were community‐based cross‐sectional studies.

**Table 1:** Summary of study characteristics in the systematic review and meta‐analysis.

Author, year	Study area/Region	Study design	Sample size	Magnitude of hygiene practices (%)	Quality assessment	Interpretation of results of wash and hygienic practices
Addis et al., 2024	Dessie Zuria district, Amhara	Institutional CS	366	22.2	High	One of five households' main sources of drinking water was the river. Latrine ownership was low, and few respondents practiced handwashing after toilet visits and before feeding children.
Akiso & Laelago, 2025	Lemo district, Central Ethiopia	Community CS	402	65.0	High	Using a protected water source and a separate kitchen decreased maternal hygiene during complementary feeding, while a latrine and nearby handwashing facility with soap had a non‐significant positive impact.
Bedada et al., 2021	Robe town, Oromia	Community CS	517	55.0	Moderate	Handwashing with soap and water before feeding children, preparing food, and after latrine use was infrequent.
Birdida et al., 2024	Borecha Woreda, Oromia	Community CS	536	34.7	High	The lack of a hand‐washing facility near the latrine and poor knowledge of hygiene practices significantly increase the difficulty of keeping maternal hygiene during complementary feeding.
Birhanu et al., 2023	Dedo district, Oromia	Community CS	501	35.8	High	Mothers' hygiene practices during complementary feeding improved significantly with nearby handwashing facilities and shorter distances to water sources.
Demmelash et al., 2020	Bahir dar district, Amhara	Community CS	604	38.9	High	Mothers' hygiene practices during complementary feeding improved significantly due to the availability of nearby handwashing facilities and owning private latrines.
Fufa et al., 2020	Rural kebeles of Harari	Community CS	422	36.9	High	Maternal hygiene during complementary feeding is improved by handwashing facilities and hygiene practice training, but not by the presence of a separate kitchen.
Kassie et al., 2023	Wolita Sodo town, southern Ethiopia	Community CS	602	42.0	Moderate	Positive attitudes significantly improve hygiene in complementary feeding. Latrine access may also enhance these practices, although the effect was not statistically significant.
Tadegew et al., 2024	Antsokia Gemza district, Amhara	Community CS	391	45.3	High	Access to piped water and awareness of the importance of good hygiene practices significantly enhance maternal hygiene practices during complementary feeding.
Tenna et al., 2022	Addis Ababa	Community CS	602	60,8	Moderate	Access to a water source in the residence compound and the presence of a handwashing facility after the toilet significantly enhance maternal hygiene practices during complementary feeding.
Teshome et al., 2022	Tegedie District, Tigray	Community CS	576	33.6	High	The availability of a handwashing station near the latrine, the presence of a three‐compartment dishwashing station, and a designated area for storing raw and cooked foods significantly enhance maternal hygiene during complementary feeding.
Zeleke et al., 2022	Debark town, Amhara	Community CS	423	44.9	High	Access to a piped water source, the use of a modern stove for cooking, the presence of a separate kitchen for food preparation, and the presence of a handwashing facility after the toilet significantly enhance maternal hygiene practices during complementary feeding.

Across all studies, a total of 5942 participants were included, with sample sizes ranging from 366 participants in Dessie Zuria District, Amhara Region [19], to 604 participants in Bahir Dar District, also in the Amhara Region [20]. Maternal literacy rates varied considerably across the studies, ranging from 34.9% to 94.9%. Similarly, the prevalence of good hygiene practices during complementary feeding showed substantial variation, ranging from 22.2% in Dessie Zuria, Amhara Region [19], to 62.0% in Lemo District, Central Ethiopia [24] (Table 1).

### 3.3 Critical Appraisal

Quality appraisal of included studies was conducted using the Joanna Briggs Institute (JBI) Critical Appraisal Checklist for Analytical Cross‐Sectional Studies. The quality scores of the included studies ranged from 5 to 8 out of a possible 8 points. Of the 12 studies assessed, nine were rated as high quality, while the remaining three were classified as moderate quality. No study was rated as low quality, indicating an overall satisfactory methodological quality of the evidence included in this review. The full file is provided in Supporting Material 2 (SM2).

### 3.4 Water Supply, Sanitation and Hygiene Facilities

#### 3.4.1 Drinking Water Sources and Point‐of‐Use Treatment

Of the 12 studies included in the review, nine reported participants' primary sources of drinking water, which comprised piped water, protected wells, protected springs, unprotected wells, unprotected springs, and river water [8, 16, 17, 19, 20, 21, 22, 24, 27]. Four studies identified protected water sources as the main source of drinking water without specifying the type. Across five studies, more than half of the participants relied on piped water, with coverage ranging from 52.2% to 64.0% [16, 19, 21, 22, 27]. In contrast, Demmelash et al. (2020) reported that only 8.4% of participants used piped water, while most depended on wells (33.4%), springs (39.1%), and river water (19.0%). A notably high reliance on protected water sources was reported by Teshome et al. (2022), where 98.1% of participants used protected sources. Similarly, Akiso and Laelago (2025) and Birhanu et al. (2023) found that 86.3% and 61.2% of households, respectively, obtained drinking water from protected sources. Furthermore, studies by Addis et al. (2024) and Demmelash et al. (2020) reported that approximately one‐fifth of participants used river water as their primary drinking water source.

**Figure 2:**
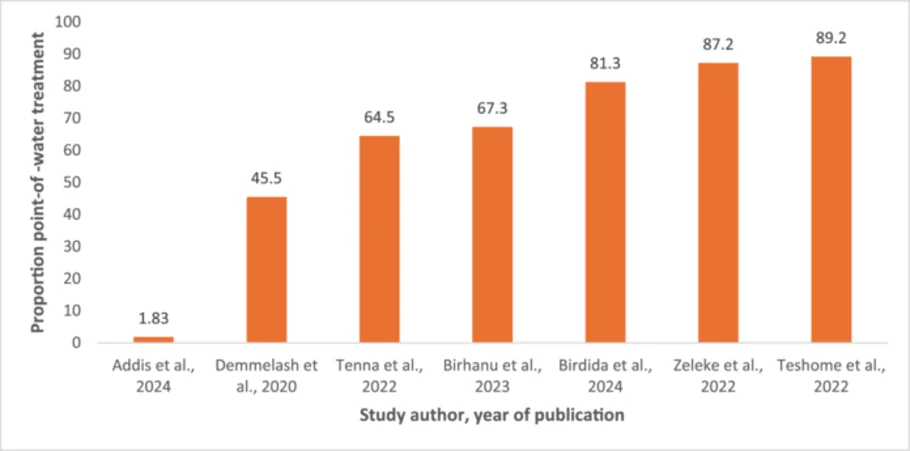
Point‐of‐use water treatment techniques employed by participants in the included studies.

Seven studies also assessed household point‐of‐use water treatment practices [8, 16, 17, 19, 20, 22, 27]. The reported treatment methods primarily included boiling and chlorination, although the prevalence varied considerably across settings. The lowest proportion of households treating drinking water was reported by Addis et al. (2024) in Dessie Zuria District, South Wollo Zone, Amhara Region, where only 1.83% practised household water treatment. In contrast, Teshome et al. (2022) reported the highest prevalence of point‐of‐use water treatment in Tegedie District, north‐western Ethiopia (Figure 2).

#### 3.4.2 Access to Latrine and Handwashing Facility

**Figure 3:**
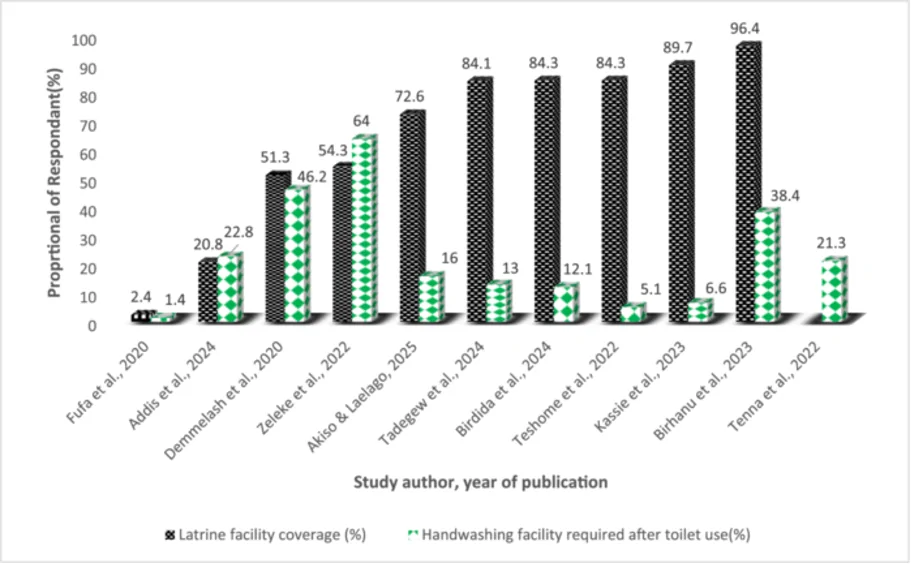
Latrine coverage and availability of handwashing facilities after toilet use.

Among the 12 studies included in this analysis, 10 reported data on latrine access, with significant variation in coverage [16, 17, 19, 20, 21, 22, 24, 25, 26]. The lowest access coverage was reported in a study conducted by Fufa et al. (2020) in rural kebeles of the Harari Region, Ethiopia. In contrast, the highest coverage was reported in a study done by Birhanu et al. (2023) in the Dedo district, Jimma zone, Oromia, Southwest Ethiopia. Data on the availability of handwashing stations needed after using toilets was additionally provided in 11 studies [16, 17, 19, 20, 21, 22, 24, 25, 26, 27], with coverage rates varying from 1.4% to 64%. Zeleke et al. (2022) reported the highest coverage in a study conducted in Debark Town, Amhara, Northwest Ethiopia, while Fufa et al. (2020) documented the lowest access percentage in rural kebeles of the Harari Region, Ethiopia (Figure 3).

#### 3.4.3 Handwashing Practices

Handwashing practices were reported in 10 of the 12 included studies, most of which used categorical response options such as *yes/no or always/sometimes/never* [8, 16, 17, 19, 20, 21, 22, 23, 25, 26]. The assessments commonly examined handwashing behavior after toilet use, before food preparation, before feeding a child, and after cleaning a child's bottom, as well as whether soap and water were used. Reported levels of handwashing were generally higher when studies assessed the practice alone rather than specifically requiring the use of soap. Handwashing after toilet use was widely practised, with prevalence estimates of 99.7% in Antsokia Gemza District [16], 81.5% in Robe Town [23], and 81.3% in Debark Town [21].

However, when handwashing with both soap and water after toilet use was assessed, substantial variation was observed across studies. Six studies reported prevalence estimates ranging from 1.3% to 60.7% [8, 17, 19, 20, 22, 25]. The lowest levels were reported in Borecha Woreda, Oromia Region (1.3%), and Bahir Dar District, Amhara Region (9.3%) [20, 22], whereas the highest were recorded in Dessie Zuria District, Amhara Region (60.7%), and Tegedie District, north‐west Ethiopia (47.9%) [8, 19]. Intermediate levels were reported in Dedo District, south‐west Ethiopia (42.4%), and rural kebeles of the Harari Region (33.3%) [17, 25].

Similarly, the prevalence of handwashing with soap before child feeding varied considerably across settings. Studies conducted by Addis et al. (2024) and Demmelash et al. (2020) reported that 64.7% and 8.9% of participants, respectively, practised this behavior. Other studies found prevalence estimates of 32.7% in Robe Town and 89.2% in Wolaita Sodo Town [23, 26]. In Antsokia Gemza District, 99.2% of participants recognized handwashing before child feeding as necessary; however, the use of soap was not assessed [21]. These findings highlight substantial geographical variations in handwashing practices and underscore the importance of both access to hygiene resources and behavioral interventions in promoting safe complementary feeding.

#### 3.4.4 Kitchen Area Hygiene Practice

Seven studies have examined kitchen hygiene practices [8, 16, 19, 20, 22, 25, 26], focusing on aspects such as the presence of a designated food preparation area, kitchen type, the separation of cooked and raw food storage, and the proper covering of cooked food. Three studies, Birdida et al. (2024), Fufa et al. (2020), and Teshome et al. (2022), reported that 93.5%, 61.2%, and 89.2% of participants, respectively, had separate kitchens for food preparation. The findings on stove usage present a contrast: Demmelash et al. (2020) and Addis et al. (2024) found that over two‐thirds of participants reported using modern stoves (74.5% and 64.1%, respectively). However, Teshome et al. (2022) indicated a significantly lower coverage of modern stove usage, at just 33.6%.

Regarding the use of separate utensils for raw and cooked food, six of the 12 reviewed studies showed a range of practices [8, 16, 19, 20, 22, 25]. Five studies showed that most participants adhered to this hygiene measure, with compliance rates ranging from 65% to 99.1%. However, Addis et al. (2024) reported that in the Dessie Zuria district, only 28.5% of participants followed this practice. Only two out of twelve studies reported on covering cooked food. Kassie et al. (2023) found that 84.9% of study participants covered cooked food when storing it. In contrast, a study by Teshome et al. (2022) revealed that only 60.3% of participants covered their cooked food during storage.

### 3.5 Contribution of Wash in Maternal Hygiene Practice

Of the 12 studies included in this review, five studies conducted in different regions of Ethiopia have examined the relationship between access to piped or protected water sources and the hygienic practices of mothers during complementary feeding. These studies emphasize the significance of obtaining clean water from improved sources to enhance maternal hygiene practices during complementary feeding. According to the analysis, collecting water from piped/protected sources enhanced maternal hygiene practice during complementary feeding with an adjusted odds ratio (AOR) range of 1.49 to 7.10 at a 95% confidence interval.

Tadegew et al. (2024) found that mothers with access to piped water were over seven times more likely to follow proper hygiene practices during complementary feeding (AOR = 7.10, 95% CI: 3.98–12.66), representing the most significant increase in hygienic practices. Similarly, Akiso and Laelago (2025) found that mothers using protected water sources were over three times more likely to practise hygiene during complementary feeding (AOR = 3.35, 95% CI: 1.83–6.63). Tenna et al. (2022) found that using tap water had a statistically insignificant impact on hygiene practices (AOR 1.49, 95% CI: 0.919–2.42). In contrast, Zeleke et al. (2022) reported a significant improvement in hygiene practices with piped water access (AOR = 1.70, 95% CI: 1.11–2.62), suggesting a positive link between piped water and maternal hygiene behaviors.

However, findings by Addis et al. (2024) showed a contrasting result: dependence on piped water sources led to a decrease in hygiene practices (AOR = 0.44, 95% CI: 0.08–2.44). Meanwhile, reliance on healthy water significantly increased hygiene practices, with an AOR of 3.35 (95% CI: 0.78–14.38), indicating that households using healthy water were more likely to follow proper hygiene practices during complementary feeding. A 2022 study by Tenna et al. in Addis Ababa slum households indicated that having a water source on the premises increased the likelihood of practising hygiene by 1.49 times. However, this finding was not statistically significant (AOR = 1.49, 95% CI: 0.919–2.42).

The relationship between latrine availability and maternal hygiene during complementary feeding was examined in 7 out of 12 studies. Among these, five studies found that having a latrine significantly improved hygiene practices, with AOR ranging from 1.08 to 4.11 at a 95% CI. The highest increment was reported by Demmelash et al. (2020), where hygiene practices increased significantly (AOR = 4.11, 95% CI: 1.90–8.49). Another study found a notable increase (AOR = 2.45, 95% CI: 1.40–4.28). In contrast, the lowest impact was observed in Zeleke et al. (2022), which reported an AOR of 1.08 (95% CI: 0.54–1.61), followed by Kassie et al. (2023), where the increase was minimal and statistically insignificant (AOR = 1.39, 95% CI: 0.76–2.55). On the other hand, Akiso and Laelago (2025) found that the absence of latrines was associated with 72% lower hygiene practices (AOR = 0.28, 95% CI: 0.23–1.04). However, Birhanu et al. (2023) reported that despite having a latrine, hygiene practices slightly declined, though the decrease was not statistically significant (AOR = 0.54, 95% CI: 0.21–1.39).

Eight of twelve studies reported an association between the presence of a handwashing facility and maternal hygiene practices during complementary feeding. Six studies indicated that the presence of handwashing facilities increased the likelihood of mothers' hygienic practices during complementary feeding, with adjusted odds ratios ranging from 2.14 to 6.75. The highest was reported in a study conducted in the Bahir Dar Zuria District by Demmelash et al. (2020) (AOR = 6.75, 95%CI: 3.16–14.41), and the lowest was reported in a study conducted in slum households in Addis Ababa by Tenna et al. (2022) (AOR = 2.14, 95% CI: 1.26–3.64). Akiso and Laelago (2025) found that in the absence of handwashing facilities, the hygienic practices of mothers decreased by 54% (AOR = 0.46, 95% CI: 0.26–0.82). However, Birdida et al. (2024) found that the absence of handwashing facilities increased the practice insignificantly (AOR = 1.45, 95% CI: 0.81–2.58).

#### 3.5.1 Meta‐Analysis of WASH and Maternal Hygiene Practices

**Figure 4:**
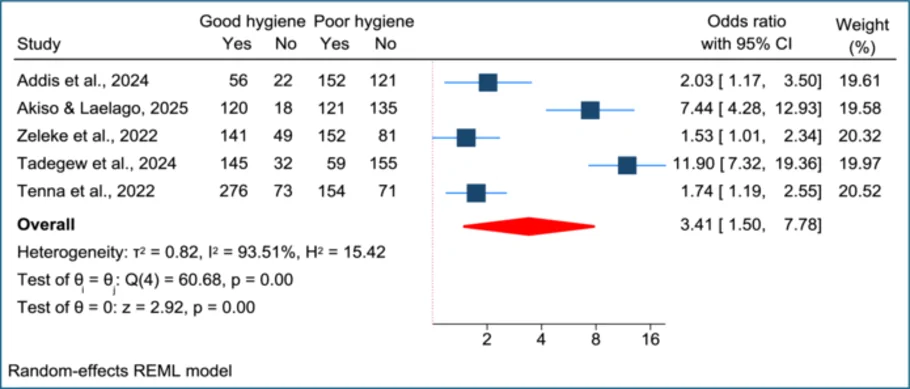
Pooled odds ratio from the meta‐analysis of drinking water sources and maternal hygiene practices during complementary feeding in Ethiopia.

This review assessed the pooled effect of using piped/protected water sources on maternal hygiene practices during complementary feeding, based on data from five studies of mothers with children under 5 years of age. In the analysis, mothers using piped or protected water sources were 3.41 times more likely to keep good maternal hygiene practices during complementary feeding compared to those using other sources, such as unprotected wells, springs, or rivers (POR = 3.41, 95% CI = 1.50, 7.78). High heterogeneity existed among the studies (*I* ^2^ = 93.51%, *p* = 0.001), with study weights ranging from 19.58% to 20.52% (Figure 4).

**Figure 5:**
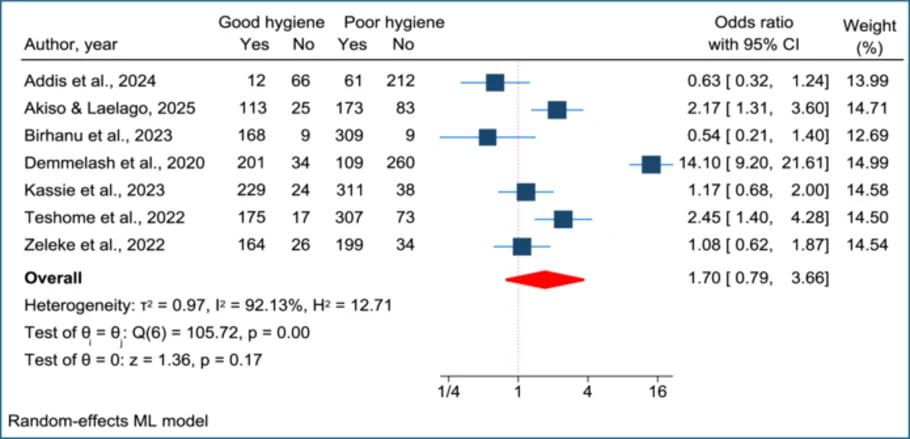
Pooled odds ratio from the meta‐analysis of latrine presence and maternal hygiene practices during complementary feeding in Ethiopia.

This meta‐analysis included seven studies reporting odds ratios from 0.54 to 14.1 for hygiene practices in households with and without latrines. The pooled results of these studies suggest that access to latrines may improve maternal hygiene during complementary feeding (POR = 1.70, 95% CI: 0.79, 3.66). The heterogeneity among the studies was high, as shown by an *I* ^2^ value of 92.13% and a p‐value of 0.001. Study weights ranged from 12.69% to 14.99% (Figure 5).

**Figure 6:**
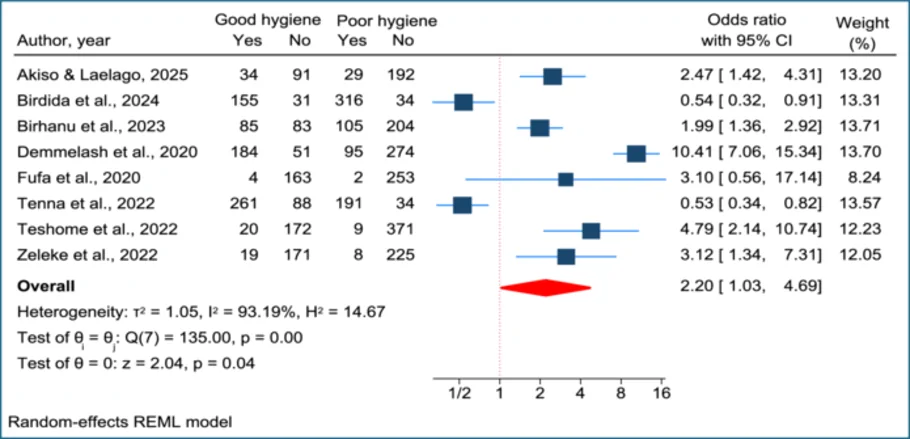
Pooled odds ratio for the association between handwashing facilities after toilet use and maternal hygiene practices during complementary feeding in Ethiopia.

This analysis of eight studies on maternal hygiene practices during complementary feeding, which compares mothers with and without handwashing facilities at latrines, found odds ratios that ranged from 0.53 to 10.41. The pooled odds ratio from these studies was 2.20 (95% CI: 1.03, 4.69), showing that handwashing facilities near latrines were likely to improve maternal hygiene during complementary feeding significantly. However, high heterogeneity was seen across studies (*I* ^2^ = 93.19%, *p* = 0.001), with individual study weights ranging from 8.24% to 13.71% (Figure 6).

### 3.6 Subgroup Analysis

Subgroup analysis by geographical region showed similar pooled effects for water supply in Northern (OR = 3.33, 95% CI: 0.92–12.01) and Central Ethiopia (OR = 3.55, 95% CI: 0.85–14.73), indicating that regional differences did not explain the substantial heterogeneity. For latrine presence, a stronger association was observed in Northern Ethiopia (OR = 2.23, 95% CI: 0.74–6.73) than in Southern Ethiopia (OR = 0.88, 95% CI: 0.42–1.83), suggesting partial regional influence on heterogeneity. For handwashing facilities, Northern Ethiopia showed the strongest association (OR = 5.79, 95% CI: 2.67–12.55), while weaker effects were observed in Central and Southern Ethiopia, indicating that regional variation may have contributed to differences in effect estimates for this WASH component (Table 2).

**Table 2:** Regional subgroup analysis of the association between WASH facilities and maternal hygiene practices during complementary feeding in Ethiopia.

WASH variables	Grouped by geographical location	Study included	Pooled OR (95% CI)	*I* ^2^ (%)	Heterogeneity *p*‐value
Water access	Northern Ethiopia	Addis et al., 2024	3.33 (0.92–12.01)	95.3%	*p* < 0.001
		Zeleke et al., 2022			
		Tadegew et al., 2024			
	Central Ethiopia	Tenna et al., 2022	3.55 (0.85–14.73)	94.4%	*p* < 0.001
		Akiso & Laelago, 2025			
	Overall	Above listed 5 studies	3.41 (1.50–7.78)	93.5%	*p* < 0.001
Latrine presence	Northern Ethiopia	Addis et al., 2024	2.23 (0.74, 6.73)	95.4%	*p* < 0.001
		Zeleke et al., 2022			
		Teshome et al., 2022			
		Akiso & Laelago, 2025			
		Demmelash et al., 2020			
	Southern Ethiopia	Birhanu et al., 2023	0.88 (0.42, 1.83)	48.2%	*p* ≈ 0.16
		Kassie et al., 2023			
	Overall	Above listed 7 studies	1.70 (0.79, 3.66)	92.1%	*p* < 0.001
Hand washing station presence	Central Ethiopia	Akiso & Laelago, 2025	1.13 (0.25–5.12)	94.5%	*p* < 0.001
		Tenna et al., 2022			
	Southern Ethiopia	Birdida et al., 2024	1.01 (0.19–5.28)	94.0%	*p* < 0.001
		Birhanu et al., 2023			
	Northern Ethiopia	Teshome et al., 2022	5.79 (2.67–12.55)	75.0%	*p* = 0.02
		Zeleke et al., 2022			
		Demmelash et al., 2020			
	Eastern Ethiopia	Fufa et al., 2020	3.10 (0.56–17.14)	NA	NA
	Overall	Above listed 8 studies	2.20 (1.03–4.69)	93.2%	*p* < 0.001

### 3.7 Sensitivity Analysis

Sensitivity analysis was performed to assess the influence of individual studies on the overall estimate of maternal hygiene practices during complementary feeding. This was done by sequentially omitting each study and seeing changes in the pooled effect size. For the water access analysis, all results remained statistically significant regardless of which study was excluded, showing that no single study had a disproportionate impact on the overall estimate. In contrast, the analysis of latrine presence and handwashing stations showed that excluding certain individual studies led to a loss of statistical significance. The inclusion of specific studies influenced overall effect estimates for these variables, underscoring their importance in the robustness of the findings (Table 3).

**Table 3:** Sensitivity analysis of included studies in this systematic review and meta‐analysis.

WASH variables	Omitted study	Number of studies included	Pooled odds ratio (95%CI)	*I* ^2^ (%)
Water access	Addis et al., 2024	4	3.87 (1.61, 9.28)	0.002
	Akiso and Laelago, 2025	4	2.82 (1.25, 6.37)	0.013
	Zeleke et al., 2022	4	4.18 (1.85, 9.41)	0.001
	Tadegew et al., 2024	4	2.47 (1.35, 4.51)	0.003
	Tenna et al., 2022	4	4.05 (1.74, 9.46)	0.001
Latrine presence	Addis et al., 2024	6	2.00 (0.88, 4.53)	0.096
	Akiso and Laelago, 2025	6	1.62 (0.67, 3.96)	0.286
	Birhanu et al., 2023	6	2.01 (0.90, 4.48)	0.087
	Demmelash et al., 2020	6	1.23 (0.81, 1.88)	0.337
	Kassie et al., 2023	6	1.81 (0.75, 4.36)	0.188
	Teshome et al., 2022	6	1.59 (0.66, 3.86)	0.302
	Zeleke et al., 2022	6	1.83 (0.76, 4.40)	0.176
Handwashing station presence	Akiso and Laelago, 2025	7	2.16 (0.96, 4.87)	0.063
	Birdida et al., 2024	7	2.72 (1.35, 5.45)	0.005
	Birhanu et al., 2023	7	2.23 (0.99, 5.06)	0.054
	Demmelash et al., 2020	7	1.67 (0.89, 3.14)	0.112
	Fufa et al., 2020	7	2.13 (0.99, 4.59)	0.054
	Tenna et al., 2022	7	2.74 (1.38, 5.45)	0.004
	Teshome et al., 2022	7	1.97 (0.91, 4.27)	0.086
	Zeleke et al., 2022	7	2.09 (0.94, 4.65)	0.069

## 4 Discussion

This systematic review and meta‐analysis demonstrated that WASH facilities play a critical role in promoting maternal hygiene practices during complementary feeding in Ethiopia. Consistent with findings from other African settings, access to safe water and handwashing facilities was associated with improved feeding hygiene practices among mothers [28, 29]. Although substantial heterogeneity was observed across the included studies, this likely reflects variations in geographical settings, population characteristics, levels of WASH access and methodological approaches. Given the diverse socioeconomic and cultural contexts across Ethiopia, such heterogeneity is not unexpected. Nevertheless, the generally consistent direction of the associations supports the robustness of the findings and underscores the importance of strengthening WASH services to improve hygiene practices during the complementary feeding period.

Maternal education is widely recognized as a fundamental determinant of child health and hygiene behaviors, influencing caregivers' ability to understand, adopt, and sustain recommended complementary feeding practices. Education enhances mothers' capacity to access and interpret health information, make informed decisions regarding food hygiene and child nutrition, and recognize behaviors that reduce the risk of foodborne infections. Consequently, mothers with greater educational attainment are often better positioned to implement appropriate hygiene measures throughout food preparation and feeding. Conversely, limited education may restrict opportunities to acquire essential health knowledge and can intersect with broader socioeconomic disadvantages, including reduced access to safe water, sanitation facilities, and other resources required to maintain hygienic practices. These findings reinforce the importance of integrating hygiene promotion with maternal education and health literacy initiatives. Strengthening women's education, alongside targeted behavior‐change interventions, may contribute to sustainable improvements in complementary feeding hygiene and, ultimately, better child health and nutritional outcomes [30].

The studies included in this review show that different populations have different levels of household water access. Access to piped water varied greatly; in five studies, 52.2–64.0% of families used it, but Demmelash et al. (2020) found that only 8.4% did so, with most depending on potentially contaminated sources such as wells, rivers, or springs. Access to protected sources was reported to be 98.1%, 86.3%, and 61.2%. Access to safe water is fundamental to maintaining appropriate hygiene practices during complementary feeding. Although the reviewed studies indicate progress in the availability of piped and protected water sources, substantial geographical inequalities persist, with some households continuing to rely on potentially contaminated sources such as rivers, wells, and springs. Similar disparities have been reported across other low‐ and middle‐income countries [31]. Limited access to safe water can hinder hygienic food preparation, utensil cleaning, and handwashing practices, thereby increasing the risk of food contamination and exposure to enteric pathogens. Consequently, inadequate water access remains a significant barrier to optimal complementary feeding practices and may contribute to diarrheal disease and environmental enteric dysfunction, both of which can impair nutrient absorption and child growth [32].

The meta‐analysis demonstrated that access to piped or protected water sources is positively associated with improved maternal hygiene practices during complementary feeding. Mothers with access to safe water were significantly more likely to practise appropriate hygiene than those relying on unprotected sources, highlighting the importance of reliable water access in supporting hygienic food preparation and handling. This finding is consistent with evidence from low‐ and middle‐income countries, which has shown that water insecurity undermines safe feeding and hygiene behaviors [33]. Access to safe water facilitates essential hygiene activities, including handwashing, cleaning feeding utensils, and the hygienic preparation of complementary foods, thereby reducing the risk of food contamination and infection. These findings reinforce the critical role of WASH infrastructure in enabling safe complementary feeding practices and supporting improved child nutrition and health outcomes [29, 31].

There were regional differences in latrine coverage, according to the current review. Ten of the twelve studies included information on latrine availability, showing notable regional differences. Notably, Fufa et al. (2020) found that rural kebeles in the Harari Region had the lowest latrine coverage, highlighting long‐standing infrastructure and public health issues. In contrast, the Dedo district of Jimma Zone, Oromia Region, had the highest coverage, according to Birhanu et al. (2023). This study is consistent with a study conducted in India [34] and Somaliland [35]. This discrepancy could be caused by ineffective sanitation programs or helpful socioeconomic circumstances. Moreover, disparities in latrine access significantly affect hygiene practices, particularly concerning young children's complementary feeding. Improving WASH interventions in general enhances diets, hygiene practices, and services and is crucial for reducing malnutrition and diarrheal diseases, especially with accessible handwashing stations [36].

Poor hand hygiene during food preparation has been linked to food contamination, according to an earlier study carried out in rural Bangladesh [37]. However, having a handwashing station close to the latrine invariably encourages people to wash their hands after using the washroom, which supports the adoption of hygienic habits in daily activities, particularly when preparing food. Data on the presence of handwashing stations near toilets, a crucial part of hygiene and disease prevention, was obtained from the current review analysis of 12 studies. These coverage rates varied from a concerning 1.4%, as reported by Fufa et al. in Harari, to a significantly higher 64%, as reported by Zeleke et al. (2022) in Debark Town, Amhara Region. To guarantee fair public health outcomes, targeted, location‐specific interventions are necessary, as shown by the significant variation in handwashing facility availability across studies.

The results of this meta‐analysis show that having handwashing stations significantly enhances maternal and caregiver hygiene practices during complementary feeding. These results are in line with the pilot study by Biswas et al. (2017) in Bangladesh [38], which showed that placing handwashing stations near food preparation and consumption areas made mothers and caregivers wash their hands with soap much more often. The intervention, which included hygiene promotion in existing nutrition programs, was both feasible and well received, resulting in significant improvements in hygienic behavior. Evidence from Malawi, as reported by Chidziwisano et al. (2020), also shows that the presence of handwashing and behavioral change interventions can increase hygiene during complementary feeding [39].

It is important to separate kitchens to protect complementary food from pathogens, thereby promoting safe food preparation and effective practices. In the current analysis of studies in Ethiopia, Birdida et al. (2024), Fufa et al. (2020), and Teshome et al. (2022) reported that 93.5%, 61.2%, and 89.2% of households, respectively, had separate kitchens for food preparation. These figures suggest relatively high infrastructure coverage, though disparities remain. Comparatively, studies from Nigeria have shown similar trends, with separate kitchen coverage exceeding 62.7% among households with children under five in the Ibadan area [40], and in India, about 70.92% of urban and 51.32% of rural households used a separate kitchen for cooking [41]. Despite broad and variable coverage, the risk of cross‐contamination and respiratory exposure to cooking emissions remains, particularly for women and children in households where safe practices are not followed [42].

Modern stoves keep food from getting contaminated by lowering the amount of smoke and other particles that can settle on food while it's being prepared [43]. Their ability to keep cooking temperatures steady also kills harmful germs, which helps make food safer and cleaner. Different parts of Ethiopia use stoves in different ways. Demmelash et al. (2020) and Addis et al. (2024) found that 74.5% and 64.1% of households used modern stoves, respectively. Teshome et al. (2022), on the other hand, found that only 33.6% of households used modern stoves. These differences are probably due to local problems like how much things cost, how easy it is to get fuel, and cultural habits of the study community.

This review has several limitations. First, all included studies employed a cross‐sectional design, limiting the ability to establish causal relationships between WASH facilities and maternal hygiene practices during complementary feeding. Second, the small number of studies included in each meta‐analysis precluded a formal assessment of publication bias, which may have affected the interpretation of the findings. Third, substantial heterogeneity was observed across studies, likely due to differences in study settings, measurement methods, and the WASH indicators assessed. Furthermore, key WASH‐related variables were not consistently reported, restricting the scope of the analysis. As the included studies were conducted in selected regions of Ethiopia and were not nationally representative, the pooled estimates should be interpreted with caution. Despite these limitations, this review provides the most comprehensive synthesis to date of the contribution of WASH facilities to maternal hygiene practices during complementary feeding in Ethiopia.

## 5 Conclusion

This review identified considerable variation in access to WASH facilities across Ethiopia, alongside substantial heterogeneity among the included studies. Despite these differences, the findings consistently indicate that access to piped or protected water sources and the availability of handwashing facilities are important enablers of good maternal hygiene practices during complementary feeding. The meta‐analysis showed that mothers with access to piped or protected water sources and handwashing facilities were significantly more likely to practise appropriate hygiene, whereas the association between latrine access and hygiene practices was positive but not statistically significant. These findings suggest that while WASH infrastructure is essential, the availability of facilities alone may not be sufficient to achieve sustained behavior change. Hygiene practices are likely influenced by a combination of environmental, socioeconomic, and behavioral factors that shape caregivers' ability to adopt recommended feeding and food‐handling behaviors. Overall, the review highlights the need for integrated WASH interventions that combine improved access to safe water, sanitation, and handwashing facilities with effective hygiene education and behavior change strategies. Attention should be given to underserved rural communities, where gaps in WASH services remain most pronounced. Strengthening both WASH infrastructure and hygiene promotion programs has the potential to improve maternal hygiene practices during complementary feeding, reduce the risk of food contamination and childhood infections, and contribute to better child health and nutritional outcomes in Ethiopia. Further well‐designed studies are required to address the observed heterogeneity and to better understand the pathways through which WASH conditions influence maternal hygiene practices during complementary feeding.

## Author Contributions

**Negasa Eshete Soboksa:** conceptualization, methodology, formal analysis, data curation, resources, writing – original draft; writing – review and editing, software, visualization, funding acquisition, investigation. **Dinkinesh Begna Gudeta:** data curation, writing – review and editing, methodology. **Renay Helouise Van Wyk:** methodology, writing – review and editing, data curation. **Zoe Nomakhushe Nxusani:** writing – review and editing, writing – original draft. **Vhushavhelo Nedzingahe:** writing – review and editing, writing – original draft, project administration. **Xikombiso Gertrude Mbhenyane:** supervision, validation, writing – review and editing, resources.

## Funding

The authors have nothing to report.

## Ethics Statement

This study is a literature review and does not require ethical approval.

## Conflicts of Interest

The authors declare no conflicts of interest.

## Transparency Statement

The corresponding author (Negasa Eshete Soboksa) affirms that this manuscript is an honest, accurate, and transparent account of the study being reported; that no important aspects of the study have been omitted; and that any discrepancies from the study as planned (and, if relevant, registered) have been explained.

## Supporting information


Supporting File 1

Supporting File 2

Supporting File 3

## Data Availability

The data that support the findings of this study are available from the corresponding author upon reasonable request.
