# Supplementary material for: Contribution of Water, Sanitation, and Hygiene Facilities to Maternal Hygiene Practices During Complementary Feeding in Ethiopia: A Systematic Review and Meta‐Analysis
Source: Health Sci Rep. 2026 Aug 26;9(9):e73084. doi: 10.1002/hsr2.73084 (PMC13508023; doi:10.1002/hsr2.73084)
Supplement: Supplementary file 1 [file HSR2-9-e73084-s001.docx]

**Searching strategy**

***Supplementary file 1: Search strategy for the PubMed database***

| S.NO | Enquiry | Results |
| --- | --- | --- |
| 1. | "maternally"[All Fields] OR "maternities"[All Fields] OR "maternity"[All Fields] OR "mothers"[MeSH Terms] OR "mothers"[All Fields] OR "maternal"[All Fields] OR "caregiver's"[All Fields] OR "caregivers"[MeSH Terms] OR "caregivers"[All Fields] OR "caregiver"[All Fields] OR "caregiving"[All Fields] | 744,206 |
| 2. | "infant nutritional physiological phenomena"[MeSH Terms] OR ("infant"[All Fields] AND "nutritional"[All Fields] AND "physiological"[All Fields] AND "phenomena"[All Fields]) OR "infant nutritional physiological phenomena"[All Fields] OR ("complementary"[All Fields] AND "feeding"[All Fields]) OR "complementary feeding"[All Fields] | 80,124 |
| 3. | "water supply"[MeSH Terms] OR "water supply"[All Fields] OR "drinking water"[All Fields] OR "drinking water"[MeSH Terms] OR "water purification"[MeSH Terms] OR "water purification"[All Fields] OR "water quality"[MeSH Terms] OR "water quality"[All Fields] | 166,658 |
| 4. | "toilet facilities"[MeSH Terms] OR ("toilet"[All Fields] OR "latrine facilities"[All Fields]) OR "toilet facilities"[All Fields] OR "Dishwashing"[All Fields] | 8,192 |
| 5. | "Hygiene"[MeSH] OR "Hand Hygiene"[MeSH] OR "Handwashing"[All Fields] OR "Hygiene Promotion"[All Fields] | 49,686 |
| 6. | 3 OR 4 OR 5 | 221,212 |
| 7. | 1 AND 2 AND 6 | 453 |
| 8. | "Ethiopia"[MeSH Terms] OR "Ethiopia"[Text Word] | 38,344 |
| 9. | 7 AND 8 | 112 |
