# Supplementary material for: Contribution of Water, Sanitation, and Hygiene Facilities to Maternal Hygiene Practices During Complementary Feeding in Ethiopia: A Systematic Review and Meta‐Analysis
Source: Health Sci Rep. 2026 Aug 26;9(9):e73084. doi: 10.1002/hsr2.73084 (PMC13508023; doi:10.1002/hsr2.73084)
Supplement: Supplementary file 2 [file HSR2-9-e73084-s002.docx]

Table S2: Bridging Hygiene Gaps: The Contribution of Water, Sanitation, and Hygiene Facilities to Maternal Hygiene Practices During Complementary Feeding in Ethiopia

Correspondence: [yeroosaa@gmail.com](mailto:yeroosaa@gmail.com)

| No. | Author, publication year | Defined Eligibility Criteria | Study Subjects and Setting Described | Exposure Measured Validly/Reliably | Objective/Standard Criteria for Condition | Confounding factors | Dealing with confounding factors | Outcomes measured in a valid and reliable way | Appropriate statistical analysis | Scores (8) | Quality (high, moderate, low) |
| --- | --- | --- | --- | --- | --- | --- | --- | --- | --- | --- | --- |
| 1 | Addis *et al.,* 2024 | Yes | Yes | Yes | Yes | Yes | Yes | No | Yes | 7 | High |
| 2 | Akiso & Laelago, 2025 | Yes | Yes | Yes | Yes | Yes | Yes | No | Yes | 7 | High |
| 3 | Bedada *et al.,* 2021 | Yes | Yes | Yes | Yes | No | No | Yes | Yes | 6 | Moderate |
| 4 | Birdida *et al.,* 2024 | Yes | Yes | Yes | Yes | Yes | Yes | Yes | Yes | 7 | High |
| 5 | Birhanu *et al.,* 2023 | Yes | Yes | Yes | Yes | Yes | Yes | Yes | Yes | 8 | High |
| 6 | Demmelash *et al.,* 2020 | Yes | Yes | Yes | Yes | Yes | Yes | Yes | Yes | 8 | High |
| 7 | Fufa *et al.,* 2020 | Yes | Yes | Yes | Yes | No | No | yes | Yes | 6 | Moderate |
| 8 | Kassie *et al.,* 2023 | Yes | Yes | No | Yes | Yes | Yes | Yes | Yes | 7 | High |
| 9 | Tadegew *et al*., 2024 | Yes | Yes | Yes | Yes | Yes | Yes | Yes | Yes | 8 | High |
| 10 | Tenna *et al.,* 2022 | Yes | Yes | Yes | Yes | Yes | No | No | Yes | 6 | Moderate |
| 11 | Teshome *et al*., 2022 | Yes | Yes | Yes | Yes | Yes | Yes | Yes | Yes | 8 | High |
| 12 | Zeleke *et al.,* 2022 | Yes | Yes | Yes | Yes | Yes | Yes | Yes | Yes | 8 | High |

**References**

Addis, A. T., Dawed, Y. A., Yimer, G. M., & Adem, Y. F. (2024). Complementary food hygiene practice and associated factors among mothers with children aged 6–23 months in Dessie Zuria, South Wollo Zone, Amhara, Ethiopia, 2023. Frontiers in Nutrition, 11. https://doi.org/10.3389/fnut.2024.1465008

Akiso, D. E., & Laelago, S. (2025). Complementary feeding hygienic practices and its associated factors among mothers of children aged 6–23 months old in Central Ethiopia, 2023. Frontiers in Public Health, 13. https://doi.org/10.3389/fpubh.2025.1475315

Bedada, S., Benti, T., & Tegegne, M. (2021). Complementary Food Hygiene Practice among Mothers or Caregivers in Bale Zone, Southeast Ethiopia: A Community Based Cross-Sectional Study. Journal of Food Science and Hygiene, 1(1), 26–36. https://doi.org/10.14302/issn.2835-2165.jfsh-20-3579

Birdida, R. T., Malka, E. S., Negash Kush, E., & Tolesa Alemu, F. (2024). Hygienic practice during complementary feeding and associated factors among mothers of children aged 6–24 months in Borecha Woreda, southwestern Ethiopia: a community-based cross-sectional study. Frontiers in Pediatrics, 12. https://doi.org/10.3389/fped.2024.1321558

Birhanu, M. T., Liga, A. D., & Jabir, Y. N. (2023). Practices of hygiene during complementary food feeding and associated factors among women with children aged 6−24 months in Dedo district, Southwest Ethiopia: A cross-sectional study. Health Science Reports, 6(12). https://doi.org/10.1002/hsr2.1771

Demmelash, A. A., Melese, B. D., Admasu, F. T., Bayih, E. T., & Yitbarek, G. Y. (2020). Hygienic Practice during Complementary Feeding and Associated Factors among Mothers of Children Aged 6-24 Months in Bahir Dar Zuria District, Northwest Ethiopia, 2019. Journal of Environmental and Public Health, 2020. https://doi.org/10.1155/2020/2075351

Fufa, D. D., Abhram, A., Teshome, A., Teji, K., Abera, F., Tefera, M., Yeshitila, M., Mengistu, M., Gezahegn, M., Gure, T., Worku, T., Alemayehu, T., & Egata, G. (2020). Hygienic practice of complementary food preparation and associated factors among mothers with children aged from 6 to 24 months in rural kebeles of Harari region, Ethiopia. Food Science and Technology (United States), 8(2), 34–42. https://doi.org/10.13189/fst.2020.080203

Kassie, G. A., Gebeyehu, N. A., Gesese, M. M., Chekol Abebe, E., Mengstie, M. A., Seid, M. A., Alebachew Bayih, W., Feleke, S. F., Amare Tesfa, N., Dejenie, T. A., Bantie, B., Solomon Kebede, Y., Zemene, M. A., Mengist Dessie, A., Anley, D. T., & Asmare Adella, G. (2023). Hygienic practice during complementary feeding and its associated factors among mothers/caregivers of children aged 6–24 months in Wolaita Sodo town, southern Ethiopia. SAGE Open Medicine, 11. https://doi.org/10.1177/20503121231195416

Tadegew, G., Chane, T., & Bogale, E. K. (2024). Hygienic complementary feeding practice and its associated factors among mothers having children aged 6–23 months in Antsokia Gemza district, Ethiopia: a cross-sectional survey. BMJ Public Health, 2(1), e000307. https://doi.org/10.1136/bmjph-2023-000307

Tenna, A., Tesfahun, E., & Derseh, D. (2022). Hygienic feeding practices of home prepared complementary foods and associated factors in slum households with children of age 6-24 months: a case study in Addis Ababa, Ethiopia. In J food safe & hyg (Vol. 8, Number 4).

Teshome, H. A., Yallew, W. W., Mulualem, J. A., Engdaw, G. T., & Zeleke, A. M. (2022). Complementary Food Feeding Hygiene Practice and Associated Factors among Mothers with Children Aged 6–24 Months in Tegedie District, Northwest Ethiopia: Community-Based Cross-Sectional Study. Hygiene, 2(2), 72–84. https://doi.org/10.3390/hygiene2020006

Zeleke, A. M., Bayeh, G. M., & Azene, Z. N. (2022). Hygienic practice during complementary food preparation and associated factors among mothers of children aged 6–24 months in Debark town, northwest Ethiopia, 2021: An overlooked opportunity in the nutrition and health sectors. PLoS ONE, 17(12 December). https://doi.org/10.1371/journal.pone.0275730
